# RAPID: A Rep-Seq Dataset Analysis Platform With an Integrated Antibody Database

**DOI:** 10.3389/fimmu.2021.717496

**Published:** 2021-08-13

**Authors:** Yanfang Zhang, Tianjian Chen, Huikun Zeng, Xiujia Yang, Qingxian Xu, Yanxia Zhang, Yuan Chen, Minhui Wang, Yan Zhu, Chunhong Lan, Qilong Wang, Haipei Tang, Yan Zhang, Chengrui Wang, Wenxi Xie, Cuiyu Ma, Junjie Guan, Shixin Guo, Sen Chen, Wei Yang, Lai Wei, Jian Ren, Xueqing Yu, Zhenhai Zhang

**Affiliations:** ^1^State Key Laboratory of Organ Failure Research, National Clinical Research, Center for Kidney Disease, Division of Nephrology, Nanfang Hospital, Southern Medical University, Guangzhou, China; ^2^Department of Bioinformatics, School of Basic Medical Sciences, Southern Medical University, Guangzhou, China; ^3^Center for Precision Medicine, Guangdong Provincial People’s Hospital, Guangdong Academy of Medical Sciences, Guangzhou, China; ^4^Key Laboratory of Mental Health of the Ministry of Education, Guangdong-Hong Kong-Macao Greater Bay Area Center for Brain Science and Brain-Inspired Intelligence, Southern Medical University, Guangzhou, China; ^5^Guangdong-Hong Kong Joint Laboratory on Immunological and Genetic Kidney Diseases, Guangdong Provincial People’s Hospital, Guangdong Academy of Medical Sciences, Guangzhou, China; ^6^State Key Laboratory of Oncology in South China, Cancer Center, Collaborative Innovation Center for Cancer Medicine, School of Life Sciences, Sun Yat-sen University, Guangzhou, China; ^7^Department of Nephrology, Hainan General Hospital, Haikou, China; ^8^Hainan Affiliated Hospital of Hainan Medical College, Haikou, China; ^9^State Key Laboratory of Ophthalmology, Zhongshan Ophthalmic Center, Sun Yat-Sen University, Guangzhou, China; ^10^Department of Pathology, School of Basic Medical Sciences, Southern Medical University, Guangzhou, China; ^11^Division of Nephrology, Guangdong Provincial People’s Hospital, Guangdong Academy of Medical Sciences, Guangzhou, China

**Keywords:** antibody database, Rep-Seq, comparative analysis, antibody annotation, public clone

## Abstract

The antibody repertoire is a critical component of the adaptive immune system and is believed to reflect an individual’s immune history and current immune status. Delineating the antibody repertoire has advanced our understanding of humoral immunity, facilitated antibody discovery, and showed great potential for improving the diagnosis and treatment of disease. However, no tool to date has effectively integrated big Rep-seq data and prior knowledge of functional antibodies to elucidate the remarkably diverse antibody repertoire. We developed a Rep-seq dataset Analysis Platform with an Integrated antibody Database (RAPID; https://rapid.zzhlab.org/), a free and web-based tool that allows researchers to process and analyse Rep-seq datasets. RAPID consolidates 521 WHO-recognized therapeutic antibodies, 88,059 antigen- or disease-specific antibodies, and 306 million clones extracted from 2,449 human IGH Rep-seq datasets generated from individuals with 29 different health conditions. RAPID also integrates a standardized Rep-seq dataset analysis pipeline to enable users to upload and analyse their datasets. In the process, users can also select set of existing repertoires for comparison. RAPID automatically annotates clones based on integrated therapeutic and known antibodies, and users can easily query antibodies or repertoires based on sequence or optional keywords. With its powerful analysis functions and rich set of antibody and antibody repertoire information, RAPID will benefit researchers in adaptive immune studies.

## Introduction

Antibodies (Abs), specialized immunoglobulins secreted by B cells, play a pivotal role in antigen recognition and neutralization. An antibody is composed of two identical heavy chains (IgHs) and two identical light chains (IgLs), each of which consists of variable and constant regions. The variable region of IgH, which constitutes the primary antigen-binding site, is generated by somatic recombination of variable (V), diversity (D), and joining (J) gene segments. During this joining procedure, nontemplated (N) and palindromic (P) nucleotide addition and exonuclease-mediated deletion occur at both the V-D and D-J junctions (1). Furthermore, specific antibodies undergo somatic hypermutation (SHM) in the germinal center upon antigen activation (2). These complex molecular mechanisms diversify antibodies substantially and enable the adaptive immune system to defend against a seemingly infinite array of pathogens. Theoretically, more than 10^13^ antibodies can be generated by the human adaptive immune system (3) and the entire collection of antibodies in a given individual is known as that individual’s antibody repertoire.

Traditional studies of antibodies focused on the isolation and characterization of antigen-specific monoclonal antibodies (mAbs), which are essential to understand immune responses, discover conserved epitopes, and design therapeutic agents (4). Several traditional approaches have been developed to detect mAbs, including hybridoma technology (5), B cell immortalization (6), single-cell PCR (7), and antibody display (8, 9). For example, the first fully human therapeutic antibody (adalimumab) with low immunogenicity compared to humanized and chimeric antibodies was discovered by phage display in 1997 (10). To bypass the laborious screening procedure to determine antigen specificity, Reddy *et al.* isolated mAbs by pairing the most abundant variable regions of IgH and IgL captured from high-throughput antibody repertoire sequencing (termed Rep-seq) (11). In contrast to traditional technologies, Rep-seq can capture millions of antibodies in a single run and allows researchers to elucidate the antibody repertoire in a comprehensive and quantitative manner. Recently, Rep-seq has shown striking potential in investigating humoral immunity (12), isolating mAbs (13, 14), evaluating vaccines (15, 16), exploring disease pathogenesis (17), diagnosing disease (18, 19), and immunotherapy approaches (20).

These previous efforts have generated a wealth of data comprising antibodies and Rep-seq datasets, representing an invaluable resource that could be leveraged to investigate the tremendously diverse antibody repertoire. Indeed, several databases and platforms have been developed to meet the needs of antibody repertoire researchers. For example, HIV-DB (21), bNAber (22), abYsis (23), EMBLIG, IMGT/LIGM-DB (24) and Thera-SAbDab (25), have been developed to catalogue particular functional antibodies, such as broadly neutralizing HIV antibodies and therapeutic antibodies. In addition, iReceptor and OAS, which focus on unifying Rep-seq datasets, enable researchers to query sequences of interest across institutions or studies (26, 27). PIRD allows researchers to compare repertoires for annotated Rep-seq datasets with a limited number of published datasets (28). There are also several Rep-seq dataset analysis platforms, including ARGalaxy, which can process raw reads and extract repertoire features online (29), BRepertoire, which concentrates on statistical analysis (30), SONAR, which is focused on inferring antibody ontogenies (31), and IgBLAST and IMGT/HighV-QUEST, which allow V(D)J gene annotation (32, 33). However, while all these previous tools are helpful, there was no platform that integrates all known antibodies, a large number of repertoires, and a feature-rich comprehensive analysis pipeline.

Here, we present a comprehensive web-based platform, named Rep-seq dataset Analysis Platform with Integrated antibody Database (RAPID, https://rapid.zzhlab.org/), that can process Rep-seq datasets online automatically and in conjunction with systematic repertoire feature comparison and antibody clone annotation. RAPID contains 2,449 Rep-seq reference datasets comprising of more than 306 million clones, 521 therapeutic antibodies, and 88059 published functional antibodies. RAPID integrates a standardized Rep-seq dataset analysis pipeline, a comparative analysis module for repertoire features, an antibody annotation module, and a powerful antibody and repertoire query module. RAPID displays results in text and image formats that can be viewed online expediently and downloaded freely. As a user-friendly Rep-seq dataset analysis platform, RAPID will assist researchers in identifying distinct repertoire signatures and antigen-specific clones in the context of various health conditions on a large scale and thus accelerate the applications of Rep-seq.

## Materials and Methods

### Rep-Seq Dataset Collection

**Figure 1:**
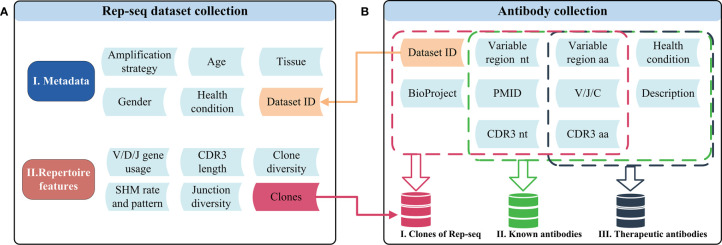
Schematic of the datasets and related features in RAPID. **(A)** Rep-seq datasets and their metadata (top) and repertoire (bottom) features. Metadata is linked with “Dataset ID” and can be obtained by it. The “Clones” in repertoire features was generated after the process of RAPID pipeline (Materials and Methods). **(B)**The antibody collections included in RAPID consist of three data sources: Rep-seq datasets, known antibodies, and therapeutic antibodies. All available information was extracted from these sources and stored. In addition, antibody sequences were analysed, and related information (such as VDJ gene usage and CDR3) were extracted and recorded. nt, nucleotide; aa, amino acid.

Rep-seq datasets included as references were either generated by our laboratory or curated from the NCBI Sequence Read Archive (SRA) database. In all, we included 592 in-house datasets produced following protocols described in the **Supplementary Materials** and 1,857 high-quality public Rep-seq datasets downloaded from the SRA database (**Supplementary Materials**). These datasets were generated *via* different amplification strategies and include samples representing different sexes, tissues, health conditions, and ages (**Figure 1A**). We processed the 2,449 Rep-seq datasets using a uniform pipeline implemented with MiXCR and in-house scripts (**Supplementary Materials**). Antibodies with the same V, J, and C genes and CDR3 nucleotide sequence (CDR3 nt) were clustered together and defined as an antibody clone. High-level features of the antibody repertoire, such as gene usage, CDR3 length, junction diversity, SHM pattern, and clone diversity were determined following the methods below (see *Repertoire Feature Extraction*) and stored in RAPID (**Figure 1A**). Thus, RAPID provides a rich source of references for the comparison of antibody repertoire features.

### Repertoire Feature Extraction

Repertoire features including V/D/J gene usage, CDR3 length distribution, junction diversity, SHM pattern, top 100 clone composition, and clone diversity for 2,449 reference Rep-seq datasets and users submitted datasets online are extracted following methods described in Yang et al. (12). Specifically, gene usage was defined as the number of clones assigned to a gene divided by the total number of clones. Similarly, the CDR3 length frequency was calculated as the number of clones of a particular length divided by the total number of clones. All clones with V/D/J assignments were included in the analysis of junction diversity. Insertion and deletion information was extracted from the column “refPoints” reported by MiXCR. Only the portions of the V gene and J gene that form the CDR3 region, (the 3’ end of V and the 5’ end of J) are included in the analysis. Both the 5’ end and 3’ end of the D gene are included. Insertions and deletions are considered mutually exclusive events, and clones containing insertions will be set to 0 when calculating deletions and vice versa. For the SHM pattern, an approach based on a position-weighted matrix is used. Firstly, clones were classified into six categories (*i.e.* IGHM, IGHD, IGHG, IGHA, IGHE, and NA) according to the “CHitsWithScore” output by MiXCR for each dataset. If C gene was not found, the isotype would be annotated as “NA”. The SHM pattern was calculated for each isotype separately and then all qualified nonredundant reads within each clone were taken into consideration. Because each clone is a basic unit in the somatic hypermutation analysis, the mutation frequency for a specific position is calculated as the sum of mutation frequencies for all mutation events (at most 3 kinds of mutation events for each position, *i.e.*, A->C, A->G, and A->T if the germline nucleotide is “A”) observed within reads supporting this clone. For this study, we only considered the region from FR1 to FR3 when measuring the mutation frequency. We investigated motif/nucleotide mutation frequencies and nucleotide transition frequencies at three different types of loci: silent loci, replacement loci, and composite loci, an approach similar to Yarri et al. (34). The mutations that happen at silent loci can only result in silent mutations; the mutations in replacement loci can only result in replacement mutations, and the composite loci contains both possibilities depending on the mutant nucleotides. The motifs we investigated in this study represent the canonical hotspots (WRCY/RGYW and WA/TW) and coldspots (SYC/GRS) reported in previous literature. The top 100 clone composition indicates the fraction of clones within top 100, which can be used to infer the clonal expansion. Clone diversity was measured using three indices: the Shannon index (Equation 1), the Simpson index (Equation 2), and D50. D50 indicates that the percentage of unique clones with accumulative reads makes up for 50% of the total. The formulas to calculate the Shannon index and Simpson index are listed below.

(1) $$\text{Shannon}\;\text{index}={\mathrm{-\Sigma}}_{i=1}^{R}p_{i}ln^{p_{i}}$$

(2) $$\text{Simpson index}=\Sigma_{i=1}^{R}p_{i}^{2}$$

where R represents the total number of clones, *i* represents the rank of a clone, and *p_i_*represents the frequency of a clone.

### Antibody Collection

The antibody collection comprises clones from the Rep-seq dataset, known antibodies, and therapeutic antibodies (**Figure 1B**). The clones in the Rep-seq dataset were derived from 7.12 billion reads representing more than 306 million clones. The dataset also includes 88,059 sequences identified from seven databases, namely: abYsis (23), bNAber (22), EMBLIG (http://acrmwww.biochem.ucl.ac.uk/abs/abybank/emblig/), HIV Molecular Immunology Database (21), IMGT/LIGM-DB (24), European Nucleotide Archive (ENA) of EMBL-EBI (35), and National Center for Biotechnology Information (NCBI) Nucleotide database (https://www.ncbi.nlm.nih.gov/nucleotide/) (**Supplementary Figure S1**). Sequences were aligned to the V/D/J germline reference by IgBLAST (32). Productive sequences were retained when they met the following two conditions: i) both V and J gene hits were obtained, and ii) unambiguous CDR3 sequences were extracted. Disease information for antibodies from EMBLIG, ENA, IMGT/LIGM-DB, and NCBI was identified using TaggerOne (version 0.2.1) (36) based on sequence descriptions and related literature titles and abstracts. The related disease for antibodies from HIV-DB and bNAber was annotated as HIV infections. The included therapeutic antibodies include 521 antibodies that were recognized by the World Health Organization (WHO) and downloaded from the Therapeutic Structure Antibody Database (Thera-SAbDab) (25). Only amino acid sequences are available for therapeutic antibodies, and regions from FR1 to FR4 and V/J genes were determined by ANARCI (37).

### Enrichment Analysis of Disease-Related Antibodies

Enrichment analysis of overlapping antigen- or disease-related antibodies was performed using a hypergeometric model implemented with the *stats.hypergeom.cdf* function within the Python package *scipy* (version 1.2.1). The false discovery rate was corrected *via* the *Benjamini-Hochberg* method implemented with a Python script.

### Development of the RAPID Web Interface

The RAPID web interface is implemented using Hyper Text Markup Language (HTML), Cascading Style Sheets (CSS), and JavaScript (JS). It is a single-page application based on the JS framework React.js while using the React component library Ant Design to unify the design style. The back end of the website uses Nginx as the HTTP and reverse proxy server, develops business logic based on Node.js, uses MySQL to manage data, and uses RabbitMQ to process the analysis task queues. Real-time notifications of task progress use WebSocket technology.

## Results

**Figure 2:**
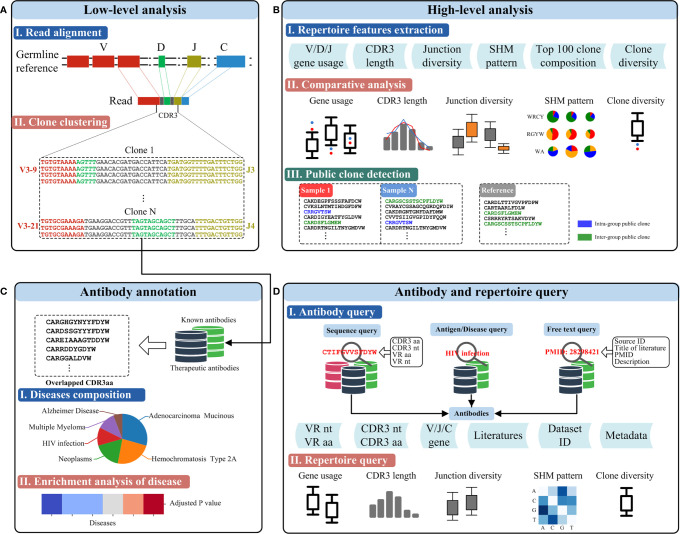
Functionalities of RAPID. **(A)** Low-level analysis. Germline genes and the antibody sequence derived from the recombination are shown schematically on top. CDR3s were identified using the RAPID bioinformatics pipeline, and clonalities of antibodies were defined according to sequence similarity and V/J gene segments (bottom). **(B)** High-level analysis of Rep-seq dataset. Repertoire features were extracted from the Rep-seq datasets (top). The features of the experimental and reference groups were compared and shown (middle). Public clones, if available, were extracted and displayed (bottom). **(C)** Antibody annotation based on CDR3 aa. Antibodies having the same amino acid CDR3 as known or therapeutic antibodies were extracted and annotated based on their matches in the database (middle). Enrichment of the annotated antibodies were analysed, and a p value was calculated. **(D)** Antibody and repertoire query function. The top panel shows several antibody queries and the schematics of the result. The bottom panel shows the visualized results of a repertoire query.

The RAPID platform builds from the availability of large Rep-seq datasets and a variety of functional antibody sequences to provide three main functionalities, including a Rep-seq dataset analysis platform (low-level analysis and high-level analysis), antibody annotation, and antibody and repertoire query (**Figure 2**).

### Rep-Seq Dataset Analysis Platform

To allow users to analyse their Rep-seq datasets rapidly, we developed a web-based automatic human IGH Rep-seq dataset analysis platform that can perform both low-level (**Figure 2A**) and high-level (**Figure 2B**) analyses. Low-level analyses, such as read alignment and clone clustering, are implemented using MiXCR because of its superb performance (38). Read alignment can identify the V/D/J/C genes and variable regions from FR1 to FR4 of each read. The isotype for each uploaded dataset can be set by users and reads aligned to this isotype would be reserved for subsequent analysis. Reads sharing the same V and J genes and CDR3 nt are clustered together as an antibody clone. Users of the RAPID platform can also customize germline reference by uploading FASTA files of V/D/J genes instead of using the platform default. High-level analyses implemented in the RAPID platform include repertoire feature extraction, comparative analysis, and public clone detection. RAPID can extract several antibody repertoire features, including V/D/J gene usage, CDR3 length, junction diversity, SHM pattern, top 100 clone composition, and clone diversity for each sample (see *Repertoire Feature Extraction*). The repertoire features of submitted samples (hereafter named the experimental group) can then be compared to references (the reference group) selected from 2,449 datasets by users. As these datasets were generated by different experimental procedures and were from heterogeneous samples, many factors might affect the repertoire comparison. For example, the location of 5’-primers used in Multiplex PCR together with read length might cause elimination of long CDR3s. To ensure users make fair comparisons and draw accurate conclusions, RAPID supports users to select references based on location of 5’- and 3’-primers, read length, sequencing platform, isotype, health condition, age, gender, and more (**Supplementary Table 2**). This is advantageous for exploring disease-associated or dynamic antibody repertoire features between different groups. Finally, clones shared by more than two samples are detected as intragroup public clones (where samples come only from the experimental group) or intergroup public clones (where samples come from both the experimental and reference groups). By virtue of these large-scale datasets, RAPID provides a powerful framework for discovering public clones that may be invaluable in pathogen clearance, disease therapy, and vaccine design. Users only need upload either single-/pair-end FASTQ or single-end FASTA files for sequencing reads, FASTA files for germline reference, and select metadata for the reference group. All of the results supplied by low-level and high-level analyses are presented in plain tabular file and image formats that can be browsed online and downloaded to a user’s local machine. The uploaded files and output results will be removed after one month.

**Figure 3:**
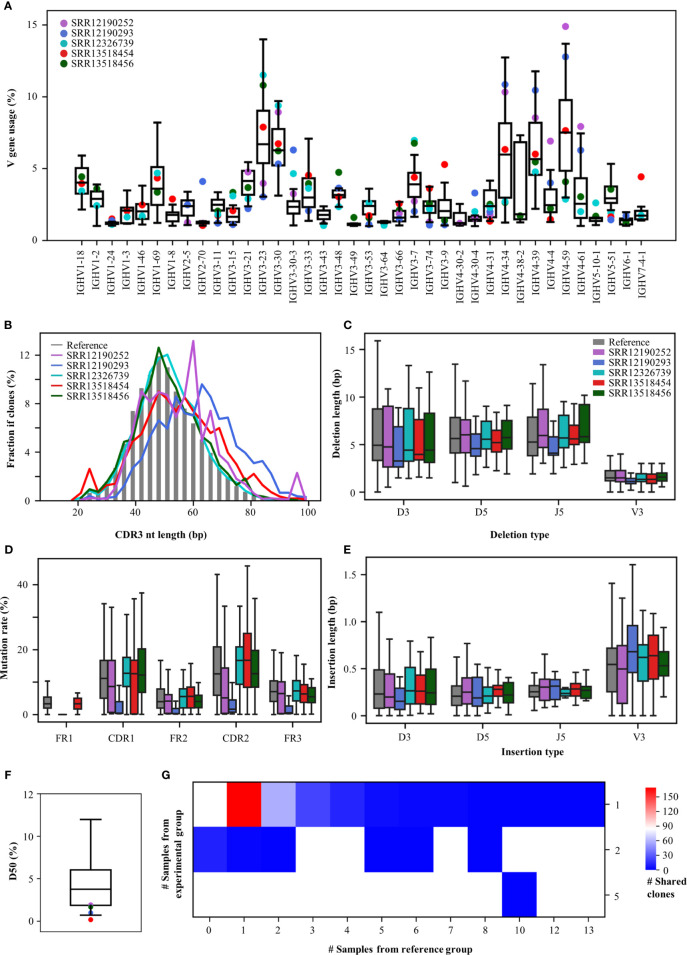
Repertoire features of COVID-19 patients compared with 32 references. **(A)** The distribution of V gene usage. The V-gene usage of the reference group is shown in the boxplot, and that of COVID-19 patients is indicated by the dots. **(B)** Length of CDR3nt sequences. The median fraction from the reference is indicated by the gray bars. The length of deletions **(C)** and insertions **(E)** at V3, D5, D3, and J5. **(D)** Mutation rates in each functional region. **(F)** The distribution of D50. **(G)** Number of shared clones. The X-axis indicates the number of references, and the Y-axis shows the number of COVID-19 samples.

To demonstrate an example usage of the Rep-seq analysis platform, we analysed the antibody repertoires generated in response to Coronavirus disease 2019 (COVID-19), which results from infection with severe acute respiratory syndrome coronavirus 2 (SARS-CoV-2). Since the start of the COVID-19 outbreak, many studies have been conducted to discover SARS-CoV-2-neutralizing antibodies (39) and to characterize the convergent signatures of T and B cell receptor repertoires for diagnosis and therapy (40, 41). We downloaded five Rep-seq datasets containing B cell receptor repertoires from COVID-19 patients from the NCBI SRA database (SRR12190252, SRR12190293, SRR12326739, SRR13518454, SRR13518456) and compared their features to those of 32 references whose Rep-seq datasets were obtained before the COVID-19 pandemic. RAPID users can select the references used in this analysis by selecting Amplification strategy as Multiplex, Tissue as PBMC, Health Condition as Healthy, 5’-primer location as FR1, 3’-primer location as CH1, Read length as 2×300 bp, and Isotype as IGHG. Although only five COVID-19 samples were analysed, the RAPID platform still identified some disease-associated repertoire signatures (**Figure 3**). For V gene usage, IGHV4-34, IGHV4-59, and IGHV4-61 increase in SRR13518454, SRR12190252, and SRR12190293. In addition, IGHV3-7 (42) and IGHV3-74 are decreased in SRR12190252 and SRR12190293 relative to the reference group (**Figure 3A**). CDR3s, as the most variable region in antibody, play important roles in determining antigen specificity. The RAPID output indicates that SRR12190252 and SRR12190293 have longer CDR3s compared to the reference groups (**Figure 3B**) (42). In addition, the COVID-19 samples have shorter deletions and longer insertions (**Figures 3C, E**) (43). SHM in the germinal center is the key process for antibody affinity maturation. In addition, we observed a higher rate of SHM in the functional region of COVID-19 samples except for SRR12190252 compared to the reference group (**Figure 3D**). Furthermore, SHM rates in SRR12326739, SRR13518454, and SRR13518456 who suffered more severe clinical pictures are strikingly higher than the other two samples. These data suggest that the SHM rate is associated with disease severity in individual patients, as has been described previously (40). Moreover, COVID-19 samples have lower D50, with obvious clonal expansion (**Figure 3F**). Importantly, 283 CDR3aa from COVID-19 samples were shared by at least one reference (**Figure 3G**). One of these CDR3aa (CARDLDYW) are shared by 13 references. Another CDR3aa, CARGFDYW, occurs in five COVID-19 samples and was shared by 10 references. Apart from the short public CDR3s, RAPID also found 20 public CDR3s whose length are longer than 48 bp. Among them, two CDR3s (CARYCSGGSCYGYYYYGMDVW, CARAGYSSSWYLDYYYGMDVW) from SRR13518456 and SRR13518454 were shared by one reference, respectively. This example demonstrates that RAPID is capable of supporting huge reference datasets and allows users to explore disease-associated repertoire signatures without resorting to expensive tools.

### Antibody Annotation

Although Rep-seq can assist researchers in capturing millions of antibody sequences at a time, it is not practical for verifying the binding specificity and functions for all of them. Thanks to advances in computational biology, several tools have been built to predict epitopes and track antibody-antigen interactions (44–46). However, these approaches are usually time-consuming and require huge computational resources, making it difficult to analyse large datasets. CDR3, as the most diverse region in antigen-binding fragments (Fabs), can serve as the primarily determinant of an antibody’s binding specificity (47). Thus, it is an ideal criterion to screen potential mAbs efficiently by searching the amino acid sequence of CDR3 (CDR3aa) from therapeutic and known antibodies (**Figure 2C**). RAPID will automatically report clones with CDR3 aa that are the same as those of therapeutic or known antibodies. The disease information of these annotated clones will also be provided. Finally, RAPID performs an enrichment analysis (see *Materials and Methods*) to discover clones whose related antigens/diseases are enriched in user-submitted samples. It should be noted that only enriched antigens/diseases whose adjusted P values are less than 0.05 will be shown.

**Figure 4:**
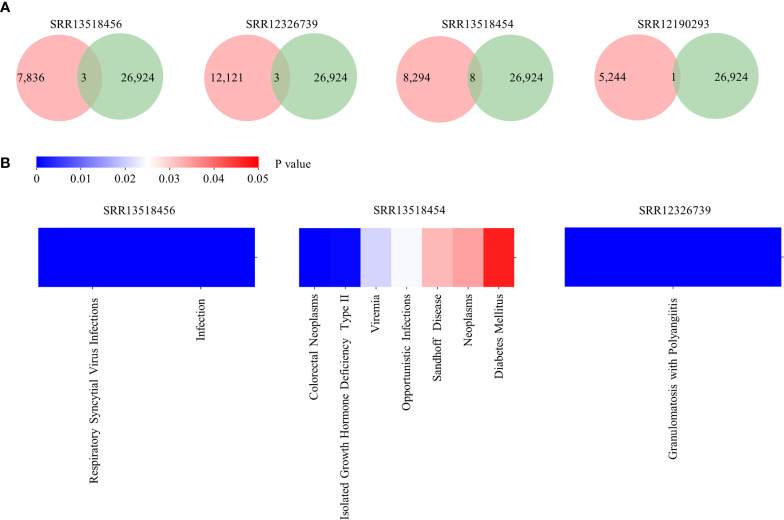
Output of Antibody annotation module. **(A)** The number of annotated clones for each sample. The pink circle represents the total clone identified in each sample and the green one indicates the number of heavy chain with detailed annotation in RAPID. **(B)** The distribution of diseases which are enriched in samples.

To demonstrate an example usage of Antibody annotation, clones identified from five COVID-19 patients were inputted. There are 3, 3, 8, and one annotated clones for SRR13518456, SRR12326739, SRR13518454, and SRR12190293, respectively (**Figure 4A**). Among them, two clones are related to Respiratory syncytial virus infections and five clones are associated to HIV infection, which suggests that these clones may be polyreactive for virus infection. Interestingly, clones related to Respiratory syncytial virus infection and Opportunistic infections were enriched in SRR13518456 and SRR13518454 (**Figure 4B**). Taken together, this annotation module can provide potential candidates for broadly neutralizing and therapeutic antibodies discovering.

### Antibody and Repertoire Query

RAPID supports online antibody and repertoire queries (**Figure 2D**). The antibody query consists of three modules: “Sequence query”, “Antigen/Disease query” and “Free text query”. The “Sequence query” module is implemented using BLAST (version 2.2.30) and search for clones from the Rep-seq dataset, therapeutic antibodies, and known antibodies. Sequence Query can output records with near-exact (identity ≥ 90%) nucleotide and exact amino acid sequence matches for the variable region and CDR3. The “Antigen/Disease query” and “Free text query” modules search for known and therapeutic antibodies. Antigen/Disease query allows users to find antigen/disease-related antibodies by directly selecting antigen/disease in the online drop-down list. Free text query enables users to query antibodies of interest by inputting source ID, title of literature, PMID, or description. For the antibody query, detailed information on resultant sequences such as V/J/C gene composition, related literature, and metadata can be accessed online by clicking hyperlinks and downloading freely. The repertoire query allows users to investigate the high-level features of repertoires by querying the Rep-seq dataset collection. Users can select samples according to metadata, and all selected samples are treated as a group. High-level features, including gene usage, CDR3 length, junction diversity, SHM pattern, clone diversity, and public clone, can be visualized online and downloaded. Several valuable signatures of the antibody repertoire can be observed by repertoire query, and these results can be used to direct subsequent work; this is analogous to conducting a pilot before an experiment is carried out.

**Figure 5:**
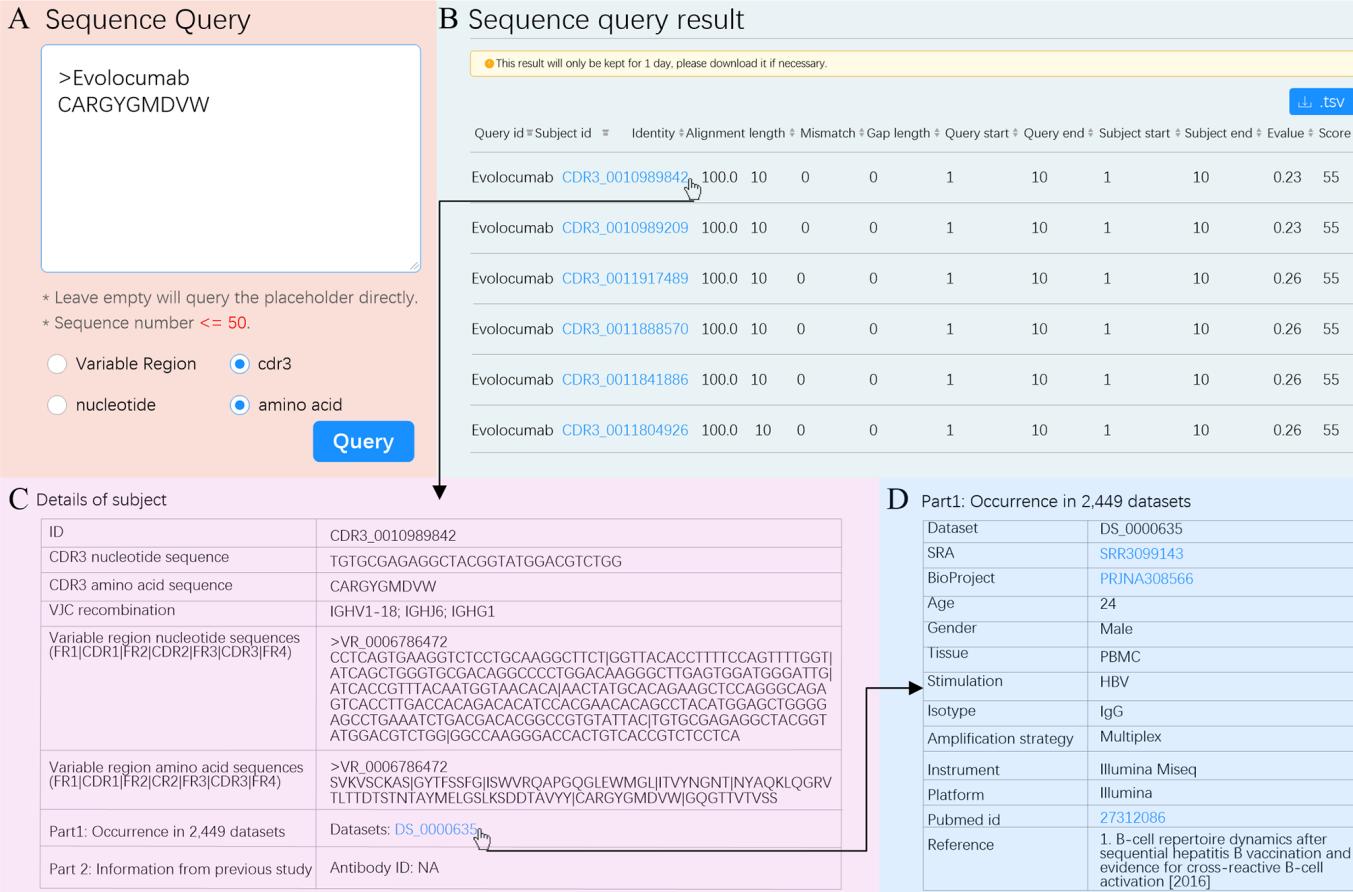
“Sequence Query” schematic. **(A)** Input options for “Sequence Query”. Selected options are marked with blue dots. **(B)** Sequence query result. The subject ID filled in blue is a hyperlink and can be clicked to see details. Hit sequences can be sorted according to any column by clicking the marker at the end of each column. **(C)** Details for subject CDR3_0010989842. **(D)** Metadata of dataset with such a subject. The accession number of SRA, BioProject, and pubmed id can be clicked to visit their original websites.

To demonstrate an example usage of Antibody query, we queried a therapeutic antibody. The first therapeutic antibody (Muromonab) was approved by United States Food and Drug Administration (US FDA) in 1986 (48). Since then, 94 therapeutic antibodies have been approved by US FDA and have become best-selling drugs (49). However, antibody discovery by experimental methods is time-consuming and difficult. The RAPID Platform allows researchers to leverage a massive antibody database to find potential therapeutic antibodies using the “Sequence query” functionality. We used Evolocumab as an example. We entered the CDR3aa of Evolocumab (CARGYGMDVW) into the text box using the cdr3 and amino acid options (**Figure 5A**). RAPID returned a total of 583 CDR3 aa with the same amino acid sequence (**Figure 5B**). For each CDR3aa, users are able to obtain details such as nucleotide sequence, V/J/C recombination, amino acid and nucleotide sequences of variable region, and accession number of dataset by clicking subject id (**Figure 5C**). The metadata of dataset, including accession number of SRA and BioProject, age, gender, tissue, stimulation, and reference, and so on can be acquired by clicking dataset id (**Figure 5D**). The information shown in **Figures 5B–D** can also be downloaded in.tsv format and used for therapeutic antibody screening experiments.

## Dissussion

B cells are an important part of the adaptive immune system, and they generate extremely diverse receptors to defeat invading pathogens. Understanding how B cell receptors recognize and neutralize antigens in humoral immunity can advance the development of clinical diagnostics and immunotherapies. Rep-seq enables immunologists to explore the entire set of these receptors—known as antibody repertoires—at unprecedented resolution, and the increased throughput of Rep-seq approaches has significantly accelerated the application of antibody repertoires. However, extracting significant characteristics from the Rep-seq dataset is crucial and challenging. To streamline the analysis of Rep-Seq datasets and advance the use of public datasets, we developed RAPID, a comprehensive Rep-Seq dataset analysis platform with an integrated antibody database. This platform has several important advantages over prior analysis tools. First, RAPID provides a user-friendly automatic analysis pipeline, incorporating low-level and high-level analyses for the Rep-Seq dataset. Second, to the best of our knowledge, RAPID contains the largest human BCR Rep-Seq database, consisting of 2,449 datasets processed by a standardized pipeline. This large reference database allows users to flexibly perform comparative analysis for their dataset. Third, it incorporates a large antibody database including 306 million clones, 521 therapeutic antibodies, and 88,059 antibodies targeting specific antigens or arising in patients with particular diseases. Based on such an expansive antibody database, RAPID supports robust antibody annotation and query services with diversified outputs.

With these rich functionalities, the RAPID platform has many practical applications. For example, it provides abundant reference datasets for comparative analysis, allowing users to examine dynamic changes in the immune repertoire between different groups. This functionality is essential for discovering biomarkers for disease diagnosis (19, 50) and for evaluating the efficacies of vaccines (15, 16). Furthermore, identifying antigen-specific neutralizing antibodies, such as those against HIV-1 (13) and SARS-CoV-2 (39), is demanding but essential for immunotherapies. With its antibody annotation and query modules, RAPID can increase efficiency and reduce the workload of antigen-specific antibody screening. Finally, public clones, which serve as ideal biomarkers of antibody convergence reflecting the canonical features of immunogens, are valuable for non-invasive disease diagnosis or prognostic surveillance (51) and for monitoring the immune response to infection or vaccination (52). RAPID provides 306 million highly reliable clones to ensure public clone detection, even if researchers conduct experiments with limited sample sizes.

Constructing an encyclopaedic atlas of human and model organism (such as mice) immune repertoires could complete the infrastructure for investigating the adaptive immune system and contribute to its applications in rational vaccine design and immunotherapies. Therefore, we will continue to collect Rep-seq datasets and antibody sequences including but not limited to (i) antibody Rep-seq datasets of light chains; (ii) antibody Rep-seq datasets from model organisms; and (iii) TCR Rep-seq datasets from humans and model organisms.

We believe that RAPID, with its elaborate Rep-seq datasets and antibody collections, could be a vital tool for assisting immunologists in exploring the immune repertoire and hastening its application.

## Data Availability Statement

The original contributions presented in the study are included in the article/**Supplementary Material**. Further inquiries can be directed to the corresponding authors.

## Ethics Statement

The studies involving human participants were reviewed and approved by Research Ethics Committee of Guangdong Provincial People’s Hospital. Written informed consent to participate in this study was provided by the participants’ legal guardian/next of kin.

## Author Contributions

YFZ, HZ, XJY, YXZ, YC, YZhu, CL, YZha, CW, CM, and SC performed the bioinformatics analyses. TC and QX developed the website. MW, QW, HT, WX, JG, and SG collected samples and conducted the biological experiments. CC, WY, LW, JR, XQY, and ZZ designed the project, biological experiments as well as bioinformatics analyses. YFZ, HZ, XJY, YC, JR, XQY, and ZZ co-wrote the manuscripts. All authors participated in discussions. All authors contributed to the article and approved the submitted version.

## Funding

This work was supported by the National Natural Science Foundation of China (NSFC) (31771479 to ZZ), NSFC Projects of International Cooperation and Exchanges of NSFC (61661146004 to ZZ), the Guangdong-Hong Kong-Macao-Joint Labs Program from Guangdong Science and Technology (2019B121205005 to XQY), and the Local Innovative and Research Teams Project of Guangdong Pearl River Talents Program (2017BT01S131 to ZZ).

## Conflict of Interest

The authors declare that the research was conducted in the absence of any commercial or financial relationships that could be construed as a potential conflict of interest.

## Publisher’s Note

All claims expressed in this article are solely those of the authors and do not necessarily represent those of their affiliated organizations, or those of the publisher, the editors and the reviewers. Any product that may be evaluated in this article, or claim that may be made by its manufacturer, is not guaranteed or endorsed by the publisher.
