# Supplementary material for: RAPID: A Rep-Seq Dataset Analysis Platform With an Integrated Antibody Database
Source: Front Immunol. 2021 Aug 13;12:717496. doi: 10.3389/fimmu.2021.717496 (PMC8414647; doi:10.3389/fimmu.2021.717496)
Supplement: Supplementary file 1 [file DataSheet_1.docx]

# **Supplementary Methods**

## **In-house dataset**

## **Subjects**

A total of 592 samples from peripheral blood, colorectal tumours and normal tissues, and bone marrow were collected. Of these, 472 samples were derived from healthy individuals (without recent infection events), 33 were from HBV-infected patients, 22 were from H7N9-infected patients, 15 were from colorectal cancer patients, and the other 50 samples were from patients with a variety of immune statuses, such as diabetes and graft versus host disease. Peripheral blood samples (1 ml) obtained from each volunteer were collected in EDTA-containing sterile tubes and stored at room temperature for no more than 6 hours. Peripheral blood mononuclear cells and bone marrow mononuclear cells were isolated by Ficoll-Paque density centrifugation using Lymphoprep™ solution (Axis-Shield, 1114547), and the isolated cells were lysed in RLT buffer (Qiagen) supplemented with 1% β-mercaptoethanol (Sigma) and then stored at -80°C for short-term storage. The tissues were cut into small pieces, ground with liquid nitrogen, and then lysed in RLT buffer (Qiagen) supplemented with 1% β-mercaptoethanol (Sigma) before being stored at -80°C for short-term storage. This protocol was approved by the Ethics Committee at Southern Medical University. Informed consent was obtained from all participants.

## **RNA extraction, reverse transcription, 5’RACE amplification, and next-generation sequencing procedures**

RNA purification was carried out using the RNeasy Mini Kit (Qiagen, 74106) according to the manufacturer’s instructions. The concentration of the RNA was determined using a NanoDrop 2000c Spectrophotometer (Thermo Fisher Scientific). Five hundred nanograms of RNA purified from each sample was used for cDNA synthesis with a total volume of 20 µl. cDNA was prepared using a SMARTer RACE cDNA Amplification Kit (Clontech, 634928) according to the manufacturer’s instructions. Forward primers were synthesized according to the SMARTer RACE protocol. The first 50 bp of the first constant domain (CH1) of the heavy chain (IgG) was used to design the reverse primers. We also designed an 8-11 bp barcode upstream of these primers to distinguish samples. One microliter of the reverse transcription mixture was used as a template in a 20 µl PCR reaction. Primers were used at a final concentration of 100 nM. The thermal cycling conditions were as follows: denaturation at 95°C for 3 min, 30 cycles of denaturation at 98°C for 20s, annealing of primer to DNA at 60°C for 15 s, and extension by Kapa HiFiHotStart Ready Mix (KAPA Biosystems, kk2602) at 72°C for 15s, followed by a ﬁnal extension for 5 min at 72°C. PCR products were analysed via electrophoresis using a 1.5% agarose gel, and the appropriate bands (~600 bp) were purified using the Nucleospin Gel & PCR Clean-up Kit (Macherey-Nagel, 704609.25). DNA concentration was measured using a NanoDrop 2000c Spectrophotometer (Thermo Fisher Scientific), and 400 ng of DNA was used to prepare libraries using a Universal DNA Library Prep Kit for Illumina V3 (Vazyme, ND607-01), strictly following the manufacturer’s instructions. Libraries were quantified using a Qubit 4.0 fluorometer (Thermo Fisher Scientific) and re-quantified using the KAPA qPCR Kit (KAPA Biosystems, 4824). The size of adapter-ligated DNA fragments (approximately 800 bp) was determined using a Bioanalyser 2100 system (Agilent). Libraries were subjected to either 2 × 300 bp or 2 × 250 bp paired-end sequencing using the Illumina MiSeq or NovaSeq platforms.

## **Rep-seq dataset enrolment criteria**

We searched for Bioprojects that were related to the antibody repertoire in the NCBI SRA database. We identified thirty-eight projects added prior to February 28, 2019. The datasets from the included projects were subjected to two consecutive filter processes. The first filter procedure was based entirely on sample metadata provided by SRA and corresponding papers. The criteria for this filter were:

- Homo sapiens
- Illumina platform
- Pair-end library layout
- Sequencing length >= 250
- Natural sample directly extracted from human tissues (excluding those samples derived from cell lines)
- No specific amplification
- Library source is either GENOMIC or TRANSCRIPTOME
- No spike-in sequences

The second filter procedure was based on the results when pre-processing was finished, and the criteria here consists of

- Number of productive reads for heavy chain > =10,000
- Fraction of heavy chain >= 20%

## **Uniform pipeline to process Rep-seq dataset**

Paired-end FASTQ files downloaded from SRA and generated by our laboratory were inputted into MiXCR (version 3.0.7) and run with the following parameters:

Align: *mixcr align --library my_library -t 8 -r align_log.txt R1 R2 alignments.vdjca -s hs*

Assemble: *mixcr assemble -r assemble_log.txt -OseparateByV=true -OseparateByJ=true -OseparateByC=true alignments.vdjcaclones.clna*

Export clones: *mixcr exportClones –c IGH clones.clna clones.txt*

Export Alignments: *mixcr exportAlignments -f -readIds -cloneId -vHit -vAlignment -jHit -jAlignment -cHit -cAlignment -nFeature FR1 -nFeature CDR1 -nFeature FR2 -nFeature CDR2 -nFeature FR3 -nFeature CDR3 -nFeature FR4 -aaFeature FR1 -aaFeature CDR1 -aaFeature FR2 -aaFeature CDR2 -aaFeature FR3 -aaFeature CDR3 -aaFeature FR4 -defaultAnchorPointsclones.clna alignments.txt*

We built germline references for V, D, J, and C gene segments locally, and the germline references for the V, D and J genes used in this study were customized using repseqio (v1.2.12, https://github.com/repseqio/repseqio). Reference sequences were obtained from IMGT/GENE DB (http://www.imgt.org/genedb/) and are provided in **Supplementary Table 1**. The formatted information for the reference constant region sequences was directly extracted from the MiXCR built-in reference (v1.5) and then appended to the formatted customized reference for V, D and J genes.
