# Supplementary material for: RAPID: A Rep-Seq Dataset Analysis Platform With an Integrated Antibody Database
Source: Front Immunol. 2021 Aug 13;12:717496. doi: 10.3389/fimmu.2021.717496 (PMC8414647; doi:10.3389/fimmu.2021.717496)
Supplement: Supplementary file 2 [file Presentation_1.pptx]

## Slide 1
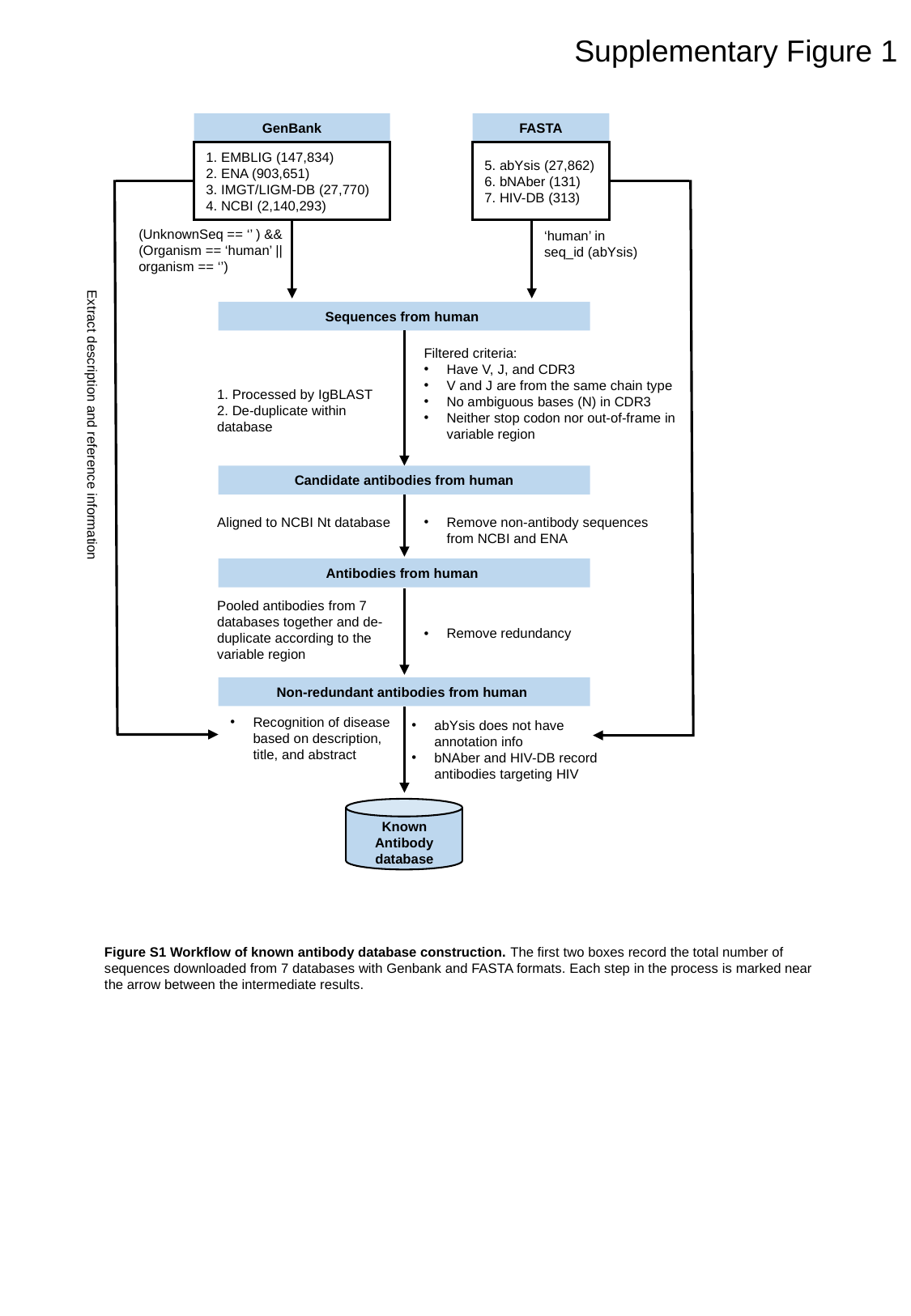

Supplementary Figure 1
GenBank
FASTA
1. EMBLIG (147,834)
2. ENA (903,651)
3. IMGT/LIGM-DB (27,770)
4. NCBI (2,140,293)
5. abYsis (27,862)
6. bNAber (131)
7. HIV-DB (313)
(UnknownSeq == ‘’ ) &&
(Organism == ‘human’ || organism == ‘’)
‘human’ in seq_id (abYsis)
Sequences from human
Filtered criteria:
Have V, J, and CDR3
V and J are from the same chain type
No ambiguous bases (N) in CDR3
Neither stop codon nor out-of-frame in variable region
1. Processed by IgBLAST
2. De-duplicate within database
Extract description and reference information
Candidate antibodies from human
Aligned to NCBI Nt database
Remove non-antibody sequences from NCBI and ENA
Antibodies from human
Pooled antibodies from 7 databases together and de-duplicate according to the variable region
Remove redundancy
Non-redundant antibodies from human
Recognition of disease based on description, title, and abstract
abYsis does not have annotation info
bNAber and HIV-DB record antibodies targeting HIV
Known
Antibody
database
Figure S1 Workflow of known antibody database construction. The first two boxes record the total number of sequences downloaded from 7 databases with Genbank and FASTA formats. Each step in the process is marked near the arrow between the intermediate results.
